# Potential Focal Adhesion Kinase Inhibitors in Management of Cancer: Therapeutic Opportunities from Herbal Medicine

**DOI:** 10.3390/ijms232113334

**Published:** 2022-11-01

**Authors:** Feiyu Chen, Zhangfeng Zhong, Cheng Zhang, Yuanjun Lu, Yau-Tuen Chan, Ning Wang, Di Zhao, Yibin Feng

**Affiliations:** 1 School of Chinese Medicine, Li Ka Shing Faculty of Medicine, The University of Hong Kong, Hong Kong SAR, China; 2 Department of Experimental Radiation Oncology, The University of Texas MD Anderson Cancer Center, 1515 Holcombe Blvd, Houston, TX 77030, USA; 3 Macau Centre for Research and Development in Chinese Medicine, Institute of Chinese Medical Sciences, University of Macau, Macau SAR, China

**Keywords:** FAK, tumor, natural compounds, Src, FAK inhibitors, metastasis

## Abstract

Focal adhesion kinase (FAK) is a multifunctional protein involved in cellular communication, integrating and transducing extracellular signals from cell-surface membrane receptors. It plays a central role intracellularly and extracellularly within the tumor microenvironment. Perturbations in FAK signaling promote tumor occurrence and development, and studies have revealed its biological behavior in tumor cell proliferation, migration, and adhesion. Herein we provide an overview of the complex biology of the FAK family members and their context-dependent nature. Next, with a focus on cancer, we highlight the activities of FAK signaling in different types of cancer and how knowledge of them is being used for screening natural compounds used in herbal medicine to fight tumor development.

## 1. Introduction

Signal communications and physical links require a constant critical balance of the intrinsic and extraneous signals in the plasma membrane. The transmitters of bidirectional communications include integrins, cytokines, growth factors, and G protein-coupled receptors [1]. Notably, integrins have been implicated to engage with extracellular matrices (ECMs) and efficiently recruit many proteins by forming intracellular complex junctions, which are well known to be focal adhesions or focal contacts [1]. Within this complex, a variety of proteins are engaged and integrated, including adaptor proteins (e.g., p130Cas, Crk), cytoskeletal proteins (e.g., paxillin, vinculin, talin), Rho family small GTPases (e.g., Rac, Rho, Cdc42), and non-receptor tyrosine kinases (e.g., focal adhesion kinase [FAK], Src family kinases) [1,2,3,4].

The dynamic assembly and disassembly of focal adhesions in response to microenvironmental stimulators are implicated to have a role in cell movement. Among all the proteins in the complex, FAK is indispensable for the conformation of focal adhesion [4]. Activation of FAK and interaction of it with integrins enable signal transmission and cytoskeletal reorganization, which contribute extensively to efficient cell motility, adhesion, and survival and maintain cell vitality [2,3].

In contrast, malfunction of the FAK signaling pathway results in dysregulation of cell behavior [2,3]. Researchers demonstrated FAK gene amplification and hyperactivation in many cancer cell types [5,6,7]. In particular, FAK itself does not act as an oncogene, and it was initially identified as a substrate of the viral proto-oncogene Src [4]. Likewise, by interacting with integrins and other transmission receptors, FAK serves as a point of convergence for many outside-in and inside-out pathways and integrates and transduces reorganized signals in cancer cells, thereby inducing malignant growth and metastasis [2,3]. Consequently, FAK is suggested to be a potential target for cancer therapeutics.

Pharmaceutical companies have recognized the value of FAK inhibitors and developed them for cancer treatment. A number of small-molecule FAK inhibitors have already undergone testing in clinical trials by different pharmaceutical companies, such as Pfizer Inc., GlaxoSmithKline, and Novartis. Currently, 12 FAK inhibitors (e.g., PF-562,271, TAE226, Y15) are in different phases of clinical trials or has completed the preclinical trials [8]. Given that herbal medicines have proven to be effective for the treatment of many diseases, including cancer [9,10,11,12,13,14,15,16,17], natural compounds from herbal medicines provide opportunities for development of new drugs [18,19], which points out a new window for the development of novel FAK inhibitors.

The composition and structure of FAK and the considerable role for FAK signaling in cellular homeostasis and development of diseases including cancer have been described thoroughly in the literature [1,2,3,4]. In the present review, we focus on the roles of FAK in major cellular components and its implications regarding cancer development and treatment. We first present an overview of FAK activation and phosphorylation and then summarize details about formation of the FAK/Src complex and its associated signaling. In addition, we review the relationship of FAK with tumor metastasis and progression and highlight the potential therapeutic opportunities for tumor suppression using natural compounds. In this context, we highlight the latest developments that how FAK is affected by exposure to natural compounds from herbal medicine to suppress tumor cell metastasis and invasion. Finally, we summarize with comments on the current FAK inhibitors and conclude about the potential use of FAK inhibitors from natural compounds to prevent tumor growth. In this review, we wish to draw the attention of researchers to the use of natural products as FAK inhibitors.

## 2. Fak Activation

**Figure 1:**
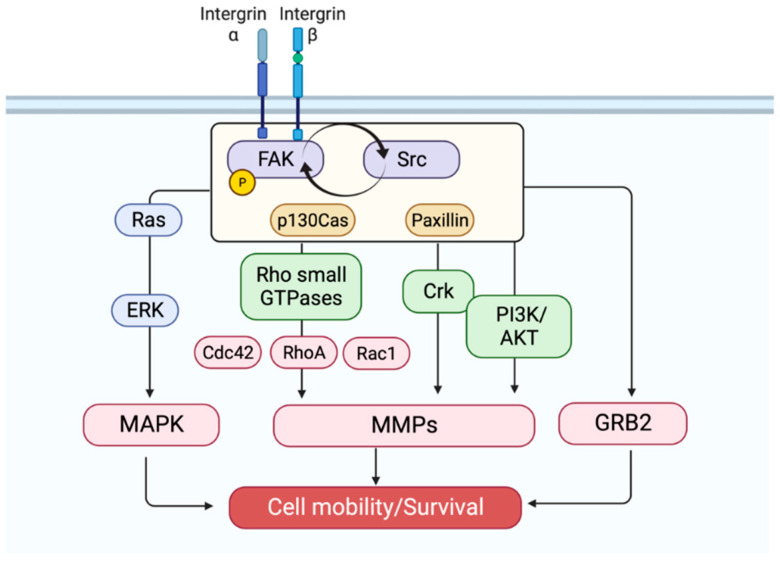
Overview of FAK regulation in focal contacts. The activation of FAK in response to integrin engagement causes the formation of phosphotyrosine-binding regions such as SH2 and SH3 domains for a number of molecules. FAK contains at least six tyrosine phosphorylation sites, including Tyr397, Tyr407, Tyr576, Tyr577, Tyr861, and Tyr925. Of these sites, the best characterized one is Tyr397, which creates an affinity docking site that can be particularly recognized by the SH2 domain-containing protein Src kinase. The high-affinity binding of FAK with Src provides multiple phosphorylation sites or structural motifs for substrate molecules, such as p130Cas (a Crk-associated substrate) and paxillin, which lead to dynamic regulation of the actin cytoskeleton and focal adhesions. Given the fact that FAK, in response to integrins, clusters with other transmittal receptors that bind to ECMs, it can integrate signals and mediate efficient cellular processes, including cell survival, motility, invasion, and angiogenesis. MAPK, mitogen-activated protein kinase; MMPs, matrix metalloproteinases; GRB2, growth factor receptor-bound protein 2.

The structure and regulation of FAK are complicated, and details regarding them were covered in recent reviews [1,2,3,4]. Therefore, herein we present a general introduction to FAK and the most characterized FAK/Src pathway to provide a brief background on FAK (Figure 1).

FAK is a non-receptor protein tyrosine kinase that receives different extracellular signals from cell-surface membrane receptors, including integrins, growth factors, cytokines, and G protein-coupled receptors [2,20]. In most cases, the activity of FAK is dependent on integrins due to its co-localization at integrin-enriched cell adhesion sites, which are called focal contacts or focal adhesions [20,21]. The integrins make up a major family of transmembrane receptors, by which cells are linked with and respond to the ECM [1,20]. The signals from the ECM are transmitted to the intracellular compartment and trigger subsequent signaling cascades for a variety of cellular activities [22,23,24].

The activation of FAK in response to integrin engagement activates phosphotyrosine-binding regions such as Src homology 2 (SH2) and SH3 domains for a number of molecules [20,24,25]. FAK contains at least six tyrosine phosphorylation sites, consisting of Tyr397, Tyr407, Tyr576, Tyr577, Tyr861, and Tyr925. The best characterized of these sites is Tyr397, which creates an affinity docking site that can be recognized by the SH2 domain-containing protein Src kinase [4,22,24,25,26]. Additionally, Src-mediated phosphorylation of Tyr577 and Tyr576 within the FAK catalytic (enzymatic/kinase) domain is indispensable for the efficient kinase activity of FAK [27]. More importantly, recruitment of Src to the FAK/Src complex facilitates the tyrosine phosphorylation of multifarious substrate proteins, which induces the activation of multiple protein kinase cascades, thereby regulating the dynamics of cell adhesion sites [22,25,28].

## 3. Fak Signaling: A Partnership with Src

Two of the best-characterized scaffold proteins in FAK/Src-mediated tyrosine phosphorylation are paxillin and p130Cas (a Crk-associated substrate), which are enriched in focal adhesions and bind directly to FAK [2]. Src phosphorylates FAK at Tyr861, which is positively linked with the SH3-mediated binding of FAK to p130Cas. This binding is also associated with increased tyrosine phosphorylation of p130Cas to SH2-containing effectors [2]. Notably, SH2-mediated binding of Crk family adaptor proteins to p130Cas is promoted, which subsequently enhances Rac activation, lamellipodia formation, and cell migration [29]. Some investigators suggested that paxillin is critical for localizing FAK to focal adhesions by the FAK focal adhesion targeting domain [30] and that FAK/Src-mediated phosphorylation at Tyr118 and Tyr31, the primary sites of paxillin, increases SH2-mediated interaction of Crk with paxillin [31,32]. On the other hand, phosphorylated paxillin at Tyr118 can promote extracellular signal-regulated kinase (ERK) 2-induced phosphorylation of paxillin, which facilitates FAK interaction with paxillin and enhances FAK activation. Conversely, FAK/Src-induced phosphorylation of paxillin at Tyr118 facilitates ERK2 activation in focal contacts [20].

Src phosphorylates FAK at Tyr925 and creates an SH2 docking site for growth factor receptor-bound protein 2 [22,27]. This connection is one of several that enable the activation of Ras and ERK2/mitogen-activated protein kinase cascades [25]. Activation of ERK2 then facilitates FAK phosphorylation at Ser910, which is also linked with reduced paxillin interaction with FAK. Collectively, this is a dynamic cycle whereby ERK phosphorylation of paxillin generates new sites for FAK phosphorylation within focal contacts while ERK and Src phosphorylation of FAK facilitate the release of FAK from focal adhesions [26]. Figure 1 shows an overview of FAK regulation in focal contacts.

## 4. Fak: An Oncogenic Driver

As described above, the high-affinity binding of FAK to Src provides multiple phosphorylation sites or structural motifs for substrate molecules such as p130Cas and paxillin, which leads to dynamic regulation of the cytoskeleton and focal adhesions [33,34]. Given that FAK clusters with other transmittal receptors and binds to the ECM in response to integrins [2], it can integrate signals and mediate efficient cellular processes, including cell survival, motility, invasion, and angiogenesis [4,20,26].

The FAK/Src pathway stimulates cell motility through the tight coordination of the Rho family of small GTPases [35]. Among them, RhoA and Rac1 are implicated to have a role in focal adhesion assembly and adhesion-dependent tyrosine phosphorylation [35]. Failure to modulate these GTPases appropriately is consistent with the disruption of cell motility observed in FAK-deficient cells [36,37]. In addition, FAK/Src signaling that promotes cell invasion through p130Cas is implicated to involve in multiple routes and results in matrix metalloproteinase (MMP)-mediated proteolytic degradation of the ECM [25,29].

Several studies have revealed that FAK is overexpressed and hyperphosphorylated in many cancer cell types [5,6,7]. For example, FAK mRNA and protein expression levels are markedly higher in breast tumors [38], colon tumors [39,40], and colorectal liver metastases [40] than in adjacent nontumorous tissues. Furthermore, hyperphosphorylation of FAK is responsible, to a certain extent, for cancer initiation and progression, which has been clarified but is not limit to small-cell lung carcinoma [41], hepatocellular carcinoma [42], oral cancer [43], prostate cancer [44], gastric cancer [45], and neuroblastoma [46]. On the other hand, FAK plays a pivotal role in the formation of focal adhesions by binding to integrins and cytoskeleton molecules [47]. This binding can lead to increased actin polymerization and branching and then formation of cell-matrix and cell-cell connections, by which the viscoelasticity of cells is altered and cell deformability is induced [48,49].

Because FAK is hyperactive in tumors, it is increasingly believed to contribute to tumorigenicity and metastasis [37,40]. Specifically, researchers identified a critical role for FAK in the promotion of glioblastoma cell invasion and migration [50,51]. Also, in non-small lung tumors, FAK expression is higher than that in normal lung tissue and is positively correlated with increased lymph node metastasis and poor clinical prognosis [52]. Notably, the Sonic hedgehog pathway can induce liver cancer cell invasion and migration through the activation of MMP-2 and MMP-9 that are mediated by FAK/AKT signaling [53]. In addition, knockdown of FAK in hepatocellular carcinoma cells dramatically downregulates MMP-2 and MMP-9 gene expression [54]. Taken together, these studies demonstrated that FAK is a potential prognostic marker for cancer and an anticancer target.

## 5. Fak Inhibitors from Natural Sources

FAK can regulate cell motility, invasion, and survival in a kinase-dependent manner [20,55,56], which is often associated with integrin-related pathways [6]. FAK also acts as a scaffold and participates in protein-protein interactions through its kinase-independent scaffolding function, which has been implicated to have a role in normal and cancer cell self-renewal and gene transcription [6,57,58,59]. FAK is recognized to be a therapeutic target for cancer, and pharmaceutical companies have developed FAK inhibitors. Most of them are molecular inhibitors that have been tested in both preclinical and clinical trials [5,6,7]. For example, Pfizer Inc. developed PF-562,271. More details about these inhibitors were described previously [5,6,7,23].

Physicians have used herbal medicines for disease prevention and treatment in traditional medical practices for many centuries [9,10,11,12,13,14,15,16]. Over the past few decades, studies have identified a number of natural compounds extracted from herbal medicine with potential for disease therapy [11,60,61,62,63,64,65]. Botanical drugs such as sinecatechins, cascara, psyllium, and senna are approved by the US Food and Drug Administration and similar organizations as prescription or over-the-counter drugs [66]. Additionally, two plant-based drugs, sinecatechins (Veregen) and crofelemer (Mytesi), have met the conditions of the US Food and Drug Administration’s botanical drug guidance for cancer therapy [67]. Nevertheless, FAK inhibitors from natural sources remain largely unexplored. Therefore, we suggest that analysis of natural products that come from herbal medicine can result in the discovery of novel FAK inhibitors.

**Figure 2:**
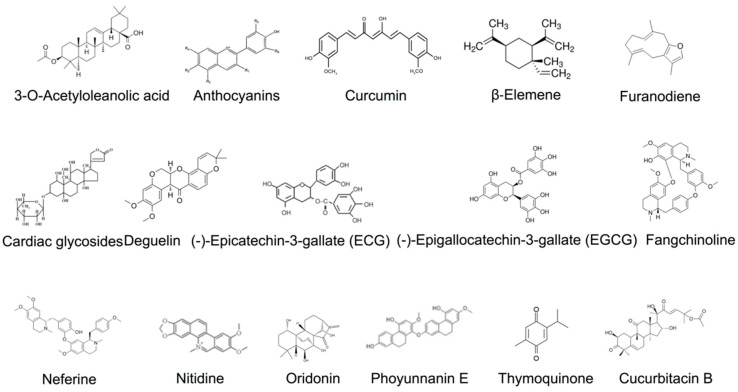
Potential FAK inhibitors from natural sources.

**Figure 3:**
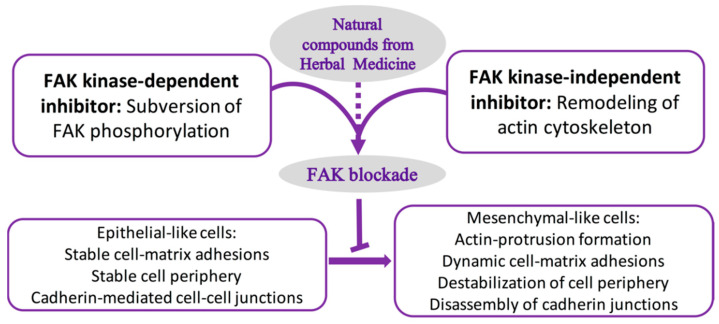
The natural compounds that potentially target FAK in cancer treatment. Natural compounds derived from Chinese herbal medicine can serve as FAK inhibitors and block the invasion and migration of cancer cells. Specifically, they can regulate cell motility, invasion, and survival in a kinase-dependent manner. Phosphorylation of FAK by Src modulates its localization and kinase activity, integrin- and E-cadherin-mediated adhesions, the formation of phosphorylation-dependent protein complexes, and cell movement and invasion. FAK also acts as a scaffold and participates in protein–protein interactions through its kinase-independent scaffolding function. These effects lead to EMT in cancer cells.

In the section below, we summarize the bioactive compounds from herbal medications with FAK-inhibitory effects that can be used to fight various tumor cell types and preclinical tumor models (Figure 2). We also discuss the recent advances in FAK research regarding prevention of cancer progression and metastasis. Furthermore, we highlight the modes of action of FAK in kinase-dependent and -independent scaffolding forms to suppress cancer cell metastasis and invasion (Table 1, Figure 3). We also highlight the roles of FAK signaling in both stromal and tumor cell biology that offer support and rationale for the use of FAK inhibitors as valuable cancer therapeutic strategies.

## 6. Fak Kinase-Dependent Inhibitors: Subversion of Fak Phosphorylation

### 6.1. 3-*O*-Acetyloleanolic Acid

3-*O*-acetyloleanolic acid (3AOA), a pentacyclic triterpenoid compound isolated from the seeds of *Vigna sinensis* K., has demonstrated potent antitumor and antiangiogenesis activity [94,95,96]. 3AOA repressed tumor growth, tumor-triggered lymphangiogenesis, and sentinel lymph node metastasis in an oral cancer sentinel lymph node animal model [68]. These inhibitory effects occur via suppression of the phosphorylation of a series of lymphangiogenesis-associated downstream factors, including FAK, AKT, phosphoinositide 3-kinase (PI3K), and ERK1/2 [68]. Therefore, 3AOA is a potential therapeutic agent for metastasis prevention and treatment in patients with oral cancer.

### 6.2. Black Rice Anthocyanins

Anthocyanins are isolated from black rice, which is a healthy food owing to its beneficial effects on the gastrointestinal tract and liver [97]. Black rice anthocyanins (BRACs) have potential pharmacological functions, such as antioxidant, anti-inflammatory, anticancer, and antimetastatic effects [98]. BRACs have demonstrated inhibitory effects on tumor progression and metastasis via cellular signal transduction [98]. Zhou et al. [69] reported that BRACs decrease cell migration distance and the number of invading breast cancer cells. Furthermore, epithelial-mesenchymal transition (EMT) is inhibited by BRACs, which also increase levels of the epithelial cell marker E-cadherin and decrease levels of the mesenchymal phenotype markers vimentin and fibronectin. In addition, phosphorylation of FAK, Src, and p130Cas is reduced after BRAC-based treatment. The interactions between FAK and Src, Src and p130Cas, and FAK and p130Cas are decreased by BRACs, as well. These findings demonstrate that BRACs inhibit the metastasis of HER-2–positive breast cancer in vitro and that the Src/FAK/p130Cas signaling pathway plays an important role in these inhibitory effects of BRACs.

### 6.3. Curcumin

The rhizome *Curcuma longa* is commonly used as spice in India and as an herbal medicine in China. A number of compounds have been extracted from some species of *Curcuma*, in which curcumin, β-elemene, and furanodiene are characteristic bioactive constituents [99,100]. Curcumin has significant therapeutic activities, including anti-inflammatory, antioxidant, antimicrobial, and anticancer activity. It can induce tumor cell apoptosis via modulation of different signaling pathways and arrest of the tumor cell cycle. However, the preventative and therapeutic functions of curcumin in metastatic tumors remain unknown.

Leu et al. [101] were the first to report the suppression of Src and FAK activity by curcumin in colon cancer cells. FAK activity can be directly inhibited by curcumin, and decreased Src activity mediated by curcumin can attenuate FAK phosphorylation, thereby repressing colon cancer metastasis. Similarly, Chen et al. [70] identified the effects of curcumin on inhibition of metastasis of colon cancer. They demonstrated that curcumin suppresses the invasion and migration of colon cancer cells and considerably reduces the number of metastatic liver nodules and growth of primary tumors in mice. Additionally, expression of CD24, a cell surface molecule, is suppressed by curcumin dose-dependently. CD24 induces Src to promote FAK phosphorylation and paxillin upregulation, which in turn increases integrin-dependent adhesion [102]. In contrast, downregulation of CD24 inhibits the interaction between CD24 and FAK and prevents the proliferation and invasion of colon cancer cells [70], suggesting that curcumin has preventative and therapeutic effects on colon cancer.

The clinical application of curcumin is hampered due to its rapid metabolism, but recent studies showed that exposure of curcumin to visible light improves its bioavailability and enhances its potential effect against cancer cell apoptosis [103,104]. Recently, Mani et al. [71] investigated the efficacy of treatment with curcumin against bladder cancer cell adhesion and migration under light exposure. They preincubated the bladder cancer cell lines RT112, UMUC3, and TCCSUP with low concentrations of curcumin and then exposed them to visible light. Compared with the use of curcumin or light alone, the combination of curcumin and light resulted in greater inhibition of tumor cell adhesion. Meanwhile, integrin subtypes were dissimilarly modified in these cell lines, and the integrins subunits α3, α5, and β1 were involved in the modulation of adhesion and migration by curcumin. Mani and colleagues revealed that integrins β1 and α5 are related to cell adhesion and migration in UMUC3 cells and that integrins β1 and α5 control chemotaxis in RT112 cells but that only the α5 subunit is involved in the cell adhesion process [71]. In summary, exposure to a low dose of curcumin plus light blocks bladder cancer cell adhesion and migration via an integrin-dependent mechanism. Notably, expression of the integrin-relevant molecule pFAK was reduced in RT112 and UMUC3 cells by treatment with curcumin and light [71]. Because a previous study clarified that integrin α3-mediated pathways (e.g., FAK/Src, FAK/PI3K/AKT) are involved in regulation of bladder cancer cell invasion and migration [105], this study by Mani et al. may partly contribute to the antimetastatic effect of the curcumin-light combination on bladder cancer.

### 6.4. β-Elemene

β-elemene, another compound from *Curcuma*, has been effective in inhibiting tumor growth [106]. Deng et al. [72] found that β-elemene considerably inhibited the invasion and migratory capacity of gastric cancer cells in vivo and in vitro. In this study, the investigators used RNA sequencing to examine differentially expressed genes in gastric cancer cells after treatment with β-elemene and found claudin-1 expression to be reduced significantly. Meanwhile, overexpression of claudin-1 reversed the repression of cell migration and invasion by β-elemene [72]. This demonstrated that β-elemene can inhibit the invasive and migratory ability of gastric cancer cells by reducing claudin-1 expression.

Claudin-1 is one of the major constituents of the tight junctions that mediate the regulation of epithelial homeostasis [107]. Researchers have identified that claudin-1 is overexpressed in tumor cells and promotes EMT in a number of cancer cell types [108,109,110,111]. In addition, treatment with an FAK inhibitor induced downregulation of claudin-1, which demonstrated that FAK regulates claudin-1 expression [72]. Of note, claudin-1 overexpression in the presence of an FAK inhibitor restores the invasive and migratory activity of gastric cancer cells [72]. Taken together, these findings suggest that FAK regulates metastatic capacity through claudin-1 and that treatment with β-elemene decreases claudin-1 expression via reduction of FAK phosphorylation. Hence, β-elemene inhibits the metastasis of gastric cancer by modulating the FAK/claudin-1 signaling pathway [72].

### 6.5. Furanodiene

Furanodiene is a major component of volatile oil isolated from *Curcuma*. It has antitumor activity against diverse types of cancer [112,113,114,115]. Zhong et al. [73] reported reduced invasion and migration capacity of breast cancer cells after furanodiene-based treatment. Integrin αV and pFAK were downregulated in these cells accordingly, demonstrating that furanodiene has joint antimetastatic action with integrin/FAK in breast cancer cells. Zhong and colleagues also observed reduced expression of pAKT, PI3K, and MMP-9 in cells after furanodiene exposure [73]. Given evidence that FAK is involved in integrin-dependent activation of PI3Ks [116,117] and that the AKT cascade can be activated by integrins and PI3K [118], these authors suggested that the integrin/FAK and PI3K/AKT pathways jointly contribute to the metastasis-inhibiting effect of furanodiene in breast cancer cases [73]. This group further investigated the antimetastatic capacity of furanodiene in combination with doxorubicin, a common chemotherapeutic drug, for breast cancer [74]. They showed that doxorubicin exposure at a nontoxic concentration can induce the invasion and migration of highly metastatic breast cancer cells. However, combination treatment with furanodiene and doxorubicin inhibits the migration and invasion of MDA-MB-231 breast cancer cells. They also reported that treatment with doxorubicin alone can increase the expression of integrin αV and induce the phosphorylation of FAK, Src, paxillin, p85, and AKT. However, combined treatment with doxorubicin and furanodiene downregulates their protein expression accordingly [74]. Therefore, the authors stated that treatment with furanodiene has the potential to improve the anticancer efficacy of doxorubicin and overcome the side effects of chemotherapy in patients with highly metastatic breast cancer [74].

### 6.6. Cardiac Glycosides

Cardiac glycosides belong to a large family of naturally occurring compounds and are clinically employed to treat congestive heart failure and atrial arrhythmia [119]. In recent decades, some naturally occurring cardiac glycosides have demonstrated activity against a number of cancers [120,121]. Ouabain, a well-known cardiac glycoside, was reported to inhibit lung cancer cell migratory behavior by suppressing FAK activity and downregulating MMP-9 and MMP-2 [122,123]. Recently, Schneider et al. [75] evaluated the anticancer effects of the cardenolides convallatoxin and digitoxigenin monodigitoxoside in four human cancer cell lines, with both compounds exhibiting inhibitory actions in all four lines. In particular, A549 lung cancer cell invasion was reduced by 90%. Notably, in line with the effects of ouabain described above, both of these compounds decreased levels of pFAK and expression of MMP-2 and MMP-9 in cancer cells while exhibiting low toxicity in normal cells [75]. Taken together, these results offer the first insight into the effects of convallatoxin and digitoxigenin monodigitoxoside on lung cancer metastasis.

### 6.7. Deguelin

Deguelin is a natural rotenoid isolated from *Derris trifoliata* Lour. and *Mundulea sericea* that has displayed antitumor properties via a variety of mechanisms for different types of cancer [124,125,126,127]. Authors also reported that it has anti-invasion and antimigration effects via different signaling pathways [128,129,130].

A recent study demonstrated that the antimigratory and anti-invasive effects of deguelin on non-small cell lung cancer are mediated by suppression of the activity of cathepsin Z and its downstream FAK/Src/paxillin pathway along with reduced phosphorylation of FAK, paxillin, and Src [76]. This demonstrated engagement of integrin in the action of cathepsin Z, which previous studies have also shown [131,132,133]. Integrin β3 is known to be physically relevant to cathepsin Z, and the interaction of Integrin β3 and cathepsin Z is suppressed by deguelin. Also, phosphorylation of Rac1 and Cdc42, two downstream molecules in the FAK/Src/paxillin signaling pathway, is decreased in lung cancer cells after deguelin-based treatment. In addition, the effect of cathepsin Z knockdown mimics that of deguelin on NCI-H1299, NCI-H23, and A549 lung cancer cell invasion and migration [76]. Collectively, these findings suggest that deguelin exerts anti-invasion and antimigration effects at least partly by disrupting the physical interaction of cathepsin Z with integrin β3 and attenuating activation of the FAK/Src/paxillin signaling cascade [76].

### 6.8. Epicatechin-3-Gallate

Epigallocatechin-3-gallate (EGCG), epigallocatechin, epicatechin-3-gallate (ECG), and epicatechin are catechins that are mainly detected in green tea [134]. ECG exhibits antioxidant and anti-inflammatory activity and reduces the risk of cardiac mortality [135]. In human lung cancer cells, ECG reverses transforming growth factor-β1-induced EMT by upregulating epithelial phenotype E-cadherin and downregulating mesenchymal phenotype fibronectin [77]. FAK is a protein tyrosine kinase that links signaling events in cells and the ECM. Huang et al. [77] explored the possible underlying mechanisms mediating the anti-invasion activity of ECG in focal contacts. They demonstrated that transforming growth factor-β1-mediated phosphorylation of FAK was reduced in A549 cells. PF573228, a specific inhibitor of the FAK signaling pathway, induced inhibition of cell invasion similar to that induced by ECG. Therefore, Huang and colleagues suggested that the suppression of invasion by ECG occurs partly via suppression of the FAK signaling pathway in A549 lung cancer cells [77] and that ECG can be administered as an effective clinical anti-EMT agent in lung cancer patients.

### 6.9. EGCG

Of the four catechins described above, EGCG is the most abundant and exhibits anticancer properties in vitro and in vivo through a variety of mechanisms [134]. Studies demonstrated EGCG to be an MMP-9 inhibitor and prevent the invasion of cancer [136,137]. Researchers from India further demonstrated downregulation of pFAK, ERK, the integrin receptors α5β1 and αvβ3, fibronectin, and vitronectin after EGCG-based treatment in MDA-MB-231 breast cancer cells. They proposed that EGCG-mediated FAK/ERK inhibition disrupts the binding activity of activator protein 1 and nuclear factor-κB, leading to dysregulation of MMP-9 gene transcription [78,79]. They also reported that ECGC decreases the levels of FAK in MCF-7 breast cancer cells, which results in blockade of MMP-2 activation and expression [80].

Chen et al. [81] showed that treatment with EGCG decreases the levels of pFAK, pSrc, snail-1, vimentin, and MMP-9 in oral squamous cell carcinoma cells in vivo and in vitro, demonstrating that it has an antimetastatic effect on this cancer. Similarly, another study demonstrated that EGCG inhibits functional invadopodia formation by inhibiting the activation of RhoA, cortactin, FAK, and Src in oral squamous cell carcinoma cells [82]. In addition, Liu et al. [83] observed a significant reduction in the number of pulmonary metastatic nodules after EGCG administration in B16-F3m melanoma-bearing BALB/c mice. Combined administration of dacarbazine and EGCG strongly repressed melanoma cell invasion and metastasis, and the underlying mechanisms of this effect were correlated with downregulation of MMP-9 and FAK. 

### 6.10. Fangchinoline

Fangchinoline is a bis-benzylisoquinoline alkaloid that was initially isolated from the dried root of *Stephania tetrandra* [138,139]. It is considered a potential therapeutic agent in clinical practice, with extensive biological antiatherosclerosis and anti-inflammatory sterilization activity, enhancement of immunity, low toxicity, and good liver protection [139]. Fangchinoline also exhibits inhibitory effects on a range of tumors through tumor promoter blockade, apoptosis induction, or cell-cycle regulation [140,141,142,143,144]. Our group explored the antitumor mechanisms of fangchinoline in hepatocellular carcinomas and demonstrated that autophagic cell death is induced by fangchinoline via the p53/sestrin 2/5′ AMP-activated protein kinase signaling pathway in hepatocellular carcinoma cells [145].

A recent study demonstrated significant inhibition of proliferation and metastasis of two melanoma cancer cell lines, A375 and A875, by fangchinoline [84]. Notably, these inhibitory effects are mediated by suppressing the phosphorylation of FAK and its downstream pathways. Moreover, the FAK inhibitor PF-562271 attenuates fangchinoline-induced inhibition of melanoma cell growth and metastasis. Therefore, the researchers in that study concluded that the inhibitory effects of fangchinoline on melanoma may be induced by suppressing phosphorylation of FAK and its downstream FAK/paxillin signaling pathway [84].

Authors also reported that the FAK pathway is involved in the antitumor action of fangchinoline in lung cancer cells [85]. Phosphorylation of ERK1/2 at Thr202 and Tyr204, mitogen-activated protein kinase kinase-1 at Ser298, and FAK at Tyr397 was dramatically decreased by fangchinoline in a dose-dependent manner, but the total expression levels for these proteins were not affected. Phosphorylation of AKT at Ser308 was also reduced by fangchinoline without a change in the total expression of AKT. Therefore, Guo and colleagues suggested that fangchinoline suppresses lung tumor growth in an FAK-dependent manner via its downstream FAK/mitogen-activated protein kinase kinase/ERK and FAK/AKT pathways. In parallel, they demonstrated that fangchinoline significantly suppresses the migratory and invasive abilities of A549 cells and reduces the expression of paxillin, MMP-9, and MMP-2 in these cells. This is consistent with the notion that FAK/paxillin interaction is associated with cell migration and invasion [146,147]. Therefore, Guo et al. [85] speculated that fangchinoline effectively represses the invasion and metastasis of A549 lung cancer cells by inhibiting the FAK/paxillin/MMP-2/MMP-9 pathway.

### 6.11. Neferine

Neferine is a bis-benzylisoquinoline alkaloid extracted from the embryos of *Nelumbo nucifera* and is reported to possess physiological activities. Researchers selected the compounds neferine A and B for the identification of novel FAK and S6K1 dual inhibitors based on high docking scores and energy interaction. Neferine A and B were identified and were subsequently validated to inhibit C6 glioma cells proliferation while exerting no side effects on adjacent normal cells at high concentrations. They also decrease the levels of pS6K1 and pFAK dose-dependently. This suggests that neferine A and B dock in S6K1 and FAK, leading to reduced phosphorylation of FAK and S6K1 enzymes. Therefore, neferine A and B are proposed to be novel inhibitors of tumor growth via dual FAK and S6K1 docking [86]. Later, researchers identified that neferine provoked autophagy and apoptosis in IMR32 cells, confirmed by pFAK and pS6K1 decay [148].

### 6.12. Nitidine

Nitidine is a benzophenanthridine alkaloid that was first derived from the root of *Zanthoxylum nitidum*. Researchers found nitidine chloride (NC) to exhibit various biological activities, including antimalarial, anti-inflammatory, antifungal, and antiangiogenesis activities [149,150,151,152]. Also, authors reported activity of NC against a number of cancers, including hepatocellular carcinoma and renal and breast cancer [87,153,154,155]. Furthermore, inhibition of renal cancer cell metastasis by NC was achieved by suppressing the AKT signaling pathway [152]. Pan et al. [87] found that NC reduces the phosphorylation of Src and FAK in breast cancer cells. Meanwhile, the activation of mitogen-activated protein kinase stimulated by platelet-derived growth factor was significantly inhibited by NC in a dose-dependent fashion. Because activation of mitogen-activated protein kinase signaling is positively correlated with the expression of MMPs, which are known to be partially responsible for tumor metastasis [156], researchers also detected the expression and enzyme activity of both MMP-9 and MMP-2 in NC-treated breast cancer cells. Decreased expression and proteolytic activity of MMP-9 and MMP-2 were observed in breast tumors after NC-based treatment, suggesting that treatment with NC at a low concentration inhibits breast cancer cell metastasis by suppressing the Src/FAK signaling pathway [87].

### 6.13. Oridonin

Oridonin, a natural diterpenoid compound derived from the medicinal herb *Rabdosia rubescens*, is an effective cytotoxic agent in treatment of a range of tumors. In recent years, many studies demonstrated that oridonin potently suppresses tumor proliferation, blocks metastasis, and induces cell autophagy and apoptosis in breast cancer cells [88,157]. Wang et al. [88] reported that oridonin exhibits inhibitory effects on the proliferation of MDA-MB-231 and MCF-7 human breast cancer cells in a time- and dose-dependent manner. Also, treatment with oridonin decreased expression of FAK and integrin β1 in MDA-MB-231 cells and reduced MMP-2 and MMP-9 activation in these cells, suggesting that inhibition of the migration and invasion of MDA-MB-231 cells by oridonin are attributed to suppression of the integrin β1/FAK pathway.

Although oridonin is effective and safe in breast cancer treatment, it has moderate to poor effects on highly aggressive breast cancers such as triple-negative breast cancer and has complex oxygenated diterpenoid scaffolding, both of which have prevented its preclinical development. Hence, the structure of oridonin has been modified to make it simpler, with more effective capacities. Oridonin derivatives have been extensively studied for the treatment of various types of human cancer, and an increasing number of investigations have focused on the development of oridonin derivatives [158,159,160,161,162].

Structural modification of oridonin via replacement of the phenyl group with 4-pyridine resulted in a novel compound, referred to as compound 56, with considerably better efficacy than unmodified oridonin in treatment of the cancer cell lines MDA-MB-231, MDA-MB-468, DU4475, and HCC1806 [89]. The investigators in that study showed that exposure to compound 56 can reduce the expression of MMP-9 and MMP-2. Treatment with compound 56 does not change the protein level for FAK but does suppress the phosphorylation of FAK at Tyr397 in HCC1806 cells. Meanwhile, expression of integrin β1 decreases after compound 56 administration in a dose-dependent fashion. Therefore, Yao and colleagues concluded that compound 56 inhibits the metastasis of HCC1806 cells via the integrin β1/FAK pathway [89]. Another group developed an active analog of oridonin, YD0514, by modifying the D-ring of oridonin [90]. They found that treatment with YD0514 dramatically inhibited the adhesion and motility of a series of metastatic breast cancer cell lines, including GILM2, GILM3, GI101, and MDA-MB-231, and, importantly, significantly inhibited the growth of xenograft metastatic breast tumors and lung metastases. The underlying mechanisms of the inhibitory effect of YD0514 on breast cancer cells occurs through reduced expression of MMP-9, MMP-2, and FAK as well as integrin family members in addition to integrin β1, so researchers speculated that YD0514 retained the antimetastatic property of oridonin and exerted its anticancer effect via inhibition of the integrin/FAK pathway [90].

### 6.14. Phoyunnanin E

Phoyunnanin E is a chemical constituent with antimalarial properties that is extracted from *Dendrobium venustum* [163]. Petpiroon et al. [91] evaluated the bioactivity of phoyunnanin E in the human non-small lung cancer cell lines A549, H292, and H460. They found that Phoyunnanin E suppresses cell motility and growth under anchorage-independent conditions and downregulates EMT-relevant proteins, including N-cadherin, snail, slug, and vimentin, thereby possessing antimigratory properties in lung cancer cells. Phoyunnanin E also decreases the expression of integrins β3 and αV, which are commonly overexpressed in highly metastatic tumor cells [1,22]. To further demonstrate the antimigratory effect of phoyunnanin E on lung cancer cells, researchers detected a series of migration-related proteins in lung cancer cells after phoyunnanin E-based treatment [91]. Phoyunnanin E slightly reduced the expression of Rho-GTP but significantly reduced the levels of pFAK and pAKT as well as the downstream effectors Rac-GTP and Cdc42. Taken together, these findings demonstrated that treatment with phoyunnanin E promotes EMT suppression in and inhibits the migration of lung cancer cells via the integrin/FAK/AKT cascade [91].

### 6.15. Thymoquinone

Thymoquinone is the main phytoactive compound of *Nigella sativa*. It has remarkable antineoplastic ability for a range of tumor types and is nontoxic to nonneoplastic cells [164,165,166,167]. Kolli-Bouhafs et al. [92] reported that thymoquinone had antimigratory and anti-invasive effects on the human glioblastoma cell lines CCF-STTG1 and U-87. Thymoquinone also reduced the expression of FAK, MMP-2, and MMP-9 in these cells, which was accompanied by decreased phosphorylation of ERK. As the FAK pathway is interacted with the ERK pathway [25] and the FAK/ERK pathway regulates the expression of MMPs in carcinoma cells of various origins [168,169], Kolli-Bouhafs and colleagues suggested that thymoquinone exhibits antimigratory and anti-invasive ability via modulation of the FAK/ERK pathway in glioblastoma cells [92].

## 7. Fak Kinase-Independent Inhibitors: Remodeling of the Actin Cytoskeleton

Tumor metastasis is a complex process, and alterations of the biomechanical properties of tumor cells determine the pathophysiology of metastasis. The actin cytoskeleton, which is the internal scaffolding of cell and consists of a multiplex network of biopolymeric molecules, principally contributes to cell deformability and mechanical deformation properties [170,171]. In recent years, many investigations have reported that cancerous cells have stronger deformability than do nonneoplastic cells, as the cytoskeleton is reorganized to produce the traction power for cell motility and the force for forward extension of the pseudo foot [172,173]. FAK plays a pivotal role in the formation of focal adhesions by binding to integrins and cytoskeleton molecules [47]. This binding can lead to increased actin polymerization and branching and then formation of cell–matrix and cell–cell connections, by which the viscoelasticity of cells is altered and cell deformability is induced [48,49].

### 7.1. Cucurbitacin B

Cucurbitacin B (CuB) is a tetracyclic triterpene compound that is widely found in plants such as *Trichosanthes kirilowii* Maximowicz and *Momordica charantia* L. [174,175,176]. Studies have demonstrated that CuB has a variety of pharmacological activities, including antiviral, anti-inflammatory, and anticancer effects [177,178]. Its anticancer capacity is achieved via inhibition of cell proliferation and metastasis and induction of apoptosis for different types of cancer cells [179,180,181]. Furthermore, CuB can affect the actin cytoskeleton by disrupting the actin filaments and microtubule structure, thereby inhibiting carcinogenesis [93,182,183,184]. Likewise, Liang et al. [93] conducted a series of in vivo and in vitro experiments and observed alteration of the actin cytoskeleton. Notably, treatment with CuB reduces the expression of FAK, F-actin, vinculin, and vimentin, which are major regulators of cytoskeletal organization and intercellular mechanical homeostasis [185]. Based on the cross-talk of integrins and FAK and integrin-mediated regulation of the activity of Rho family proteins in cell migration [186,187,188], the expression of Rho family proteins, including Rac1, RhoA, Cdc42, and integrins, is detected in breast cancer cells after CuB exposure. CuB mediates the reorganization of cytoskeletal proteins in breast cancer cells via the Rac1/Cdc42/RhoA signaling pathway, which sheds new light on the suppression of breast cancer metastasis by CuB [93]. 

### 7.2. Thymoquinone

Researchers demonstrated engagement of the FAK/ERK/MMP cascade in the anti-invasive and antimigratory effects of thymoquinone on glioblastoma cells [92]. Notably, thymoquinone induces actin cytoskeletal organization in both CCF-STTG1 and U-87 glioblastoma cells. Therefore, thymoquinone-based treatment alters focal adhesion structures, which leads to disorganization of the actin network [92]. This suggests that thymoquinone-induced morphological changes in glioblastoma cells are attributed partly to disruption of focal contacts and actin cytoskeletal organization.

## 8. Conclusions

Despite positive responses of cancer to advanced treatments, local recurrence and dissemination still occur in a significant number of patients [189]. To a certain extent, tumor initiation is caused by genetic dysfunction, mutations, or amplifications [190]. As described above, novel agents targeting the FAK pathway are promising as therapeutics for tumor suppression.

Chinese herbal medicine has emerged as a significant source of natural antitumor agents. Potentially therapeutic substances are reported in at least 10,000 of 500,000 natural plant species [191]. According to an analysis by the U.S. Food and Drug Administration, one third of new drugs come from natural products and their derivatives, and about 80% of these drugs are used for tumor management [192]. Detailed information on these approved drugs has been reviewed by experts in this field [192]. For instance, docetaxel and paclitaxel, two of the most commonly used chemotherapeutic drugs for cancer, are extracted from *Taxus brevifolia* [193,194]. Additionally, irinotecan, a chemotherapeutic medication used for the treatment of colorectal cancer, is derived from *Camptotheca acuminata* [195].

As we know, some FAK inhibitors in preclinical could not enter into clinical trial [8], such as TAE226, it shows side effects of severely altered glucose metabolism and blood glucose levels observed in animal studies. But it can be developed to synthesize molecules with a better safety profile. As for Y15, the preclinical trial shows promise but clinical trial results remain unknown right now [8]. This review brings fresh perspectives to the exploration of novel potential FAK inhibitors. As described herein, a significant number of natural ingredients and their derivatives are identified to target the FAK pathway, and they may be used to tailor more effective options for cancer treatment and prevention.

## Figures and Tables

**Table 1:** FAK inhibitors from natural sources.

Natural Compound	Herbal Source	Tumor Type	Key Findings	Reference
	FAK kinase-dependent inhibitors			
3AOA	*V. sinensis* K.	Oral cancer	3AOA suppresses tumor growth, tumor-triggered lymphangiogenesis, and sentinel lymph node metastasis by suppressing the phosphorylation of AKT, FAK, PI3K, and ERK1/2.	[68]
Anthocyanins	Black rice	HER-2–positive breast cancer	BRACs suppress the metastasis of HER-2–positive breast cancer in vitro via the Src/FAK/p130Cas pathway.	[69]
Curcumin	*Curcuma C*. *longa*	Colon cancer	Downregulation of CD24 by curcumin inhibits the interaction of CD24 with FAK and then prevents the proliferation and invasion of colon cancer cells.	[70]
		Bladder cancer	Treatment with curcumin and light blocks bladder cancer cell adhesion and migration through inhibition of integrin/pFAK signaling.	[71]
β-elemene	*Curcuma C*. *longa*	Gastric cancer	β-elemene inhibits gastric cancer cell metastasis via modulation of the FAK/claudin-1 signaling pathway.	[72]
Furanodiene	*Curcuma C*. *longa*	Breast cancer	The integrin/FAK and PI3K/AKT pathways jointly contribute to the metastasis-inhibiting effect of furanodiene in breast cancer cases.	[73]
		Highly metastatic breast cancer	Furanodiene has the potential to improve the anticancer efficacy of doxorubicin by downregulating the phosphorylation of FAK, Src, paxillin, p85, and AKT.	[74]
Cardiac glycosides (digitoxigenin monodigitoxoside and convallatoxin)	*Digitalis lanata* Ehrh. (digitoxigenin monodigitoxoside), *Convallaria majalis* L. (convallatoxin)	Lung cancer	Cardenolides decrease the expression of pFAK, MMP-9, and MMP-2 to inhibit cancer cell migratory behavior.	[75]
Deguelin	*D. trifoliata* Lour. and *M. sericea*	Lung cancer	Deguelin exerts antimigratory and anti-invasive effects partly by disrupting the physical interaction of cathepsin Z with integrin β3 and attenuating activation of the FAK/Src/paxillin-signaling cascade.	[76]
ECG	Green tea	Lung cancer	Invasion of A549 lung cancer cells is inhibited by ECG partly through inhibition of the FAK-signaling pathway.	[77]
EGCG	Green tea	Breast cancer	EGCG-induced FAK/ERK inhibition disrupts the binding activities of nuclear factor-κB and activator protein 1, leading to dysregulation of MMP-9 gene transcription.	[78,79]
		Breast cancer	Downregulation of FAK is induced by EGCG in MCF-7 breast cancer cells, which results in blockade of MMP-2 activation and expression.	[80]
		Oral squamous cell carcinoma	EGCG decreases the levels of pFAK, pSrc, snail-1, vimentin, and MMP-9 in vivo and in vitro, demonstrating the antimetastatic. effect of EGCG on oral squamous cell carcinoma.	[81]
		Oral squamous cell carcinoma	EGCG inhibits functional invadopodia formation by inhibiting the activation of RhoA, cortactin, FAK, and Src in oral squamous cell carcinomas.	[82]
		Melanoma	EGCG is correlated with inhibition of cell invasion along with downregulation of MMP-9 and FAK in melanoma cells.	[83]
Fangchinoline	*S. tetrandra*	Melanoma	The inhibitory effect of fangchinoline on melanoma may result from suppressing phosphorylation of FAK and its downstream FAK/paxillin-signaling pathway.	[84]
		Lung cancer	Fangchinoline effectively represses cell invasion and metastasis in A549 lung cancer cells by inhibiting the FAK-paxillin/MMP-2/MMP-9 pathway.	[85]
Neferine	*N. nucifera*	Glioma	Neferine A and B are proposed to be novel inhibitors of tumor growth via dual FAK and S6K1 docking.	[86]
Nitidine	*Z. nitidum*	Breast cancer	At low concentrations, NC inhibits breast cancer cell metastasis by blocking the c-Src/FAK signaling pathway.	[87]
Oridonin	*R. rubescens*	Breast cancer	The migration and invasion of MDA-MB-231 cells by oridonin may be attributed to blockade of the integrin β1/FAK pathway.	[88]
Oridonin analogs		Metastatic breast cancer	Oridonin analogs may retain the antimetastatic property of oridonin and exert anticancer effects via inhibition of the integrin/FAK pathway.	[89,90]
Phoyunnanin E	*D. venustum*	Lung cancer	Phoyunnanin E promotes EMT suppression in and inhibits the migration of lung cancer cells via the integrin/FAK/AKT cascade.	[91]
Thymoquinone	*N. sativa*	Glioblastoma	Thymoquinone exerts antimigratory and anti-invasive effects via modulation of the FAK/ERK pathway in glioblastoma cells.	[92]
	FAK kinase-independent inhibitors			
CuB	*T. kirilowii* Maximowicz and *M. charantia* L.	Breast cancer	CuB mediates the reorganization of cytoskeletal proteins in breast cancer cells via the RAC1/CDC42/RhoA signaling pathway.	[93]
Thymoquinone	*N. sativa*	Glioblastoma	Thymoquinone-induced morphological changes in glioblastoma cells are attributed, to a certain extent, to disruption of focal contacts and actin cytoskeletal organization.	[92]

## Abbreviations

3AOA: 3-*O*-acetyloleanolic acid; BRAC, black rice anthocyanin; CuB, cucurbitacin B; ECG, epicatechin-3-gallate; ECM, extracellular matrix; EGCG, epigallocatechin-3-gallate; EMT, epithelial-mesenchymal transition; ERK, extracellular signal-regulated kinase; FAK, focal adhesion kinase; MMP, matrix metalloproteinase; NC, nitidine chloride; PI3K, phosphoinositide 3-kinase; SH2, Src homology 2.
